# Prevalence and Occupational and Environmental Risk Factors of Self-Reported Asthma: Evidence from a Cross-Sectional Survey in Seven Chinese Cities

**DOI:** 10.3390/ijerph13111084

**Published:** 2016-11-04

**Authors:** Qing-Ling Fu, Yue Du, Geng Xu, Hua Zhang, Lei Cheng, Yan-Jun Wang, Dong-Dong Zhu, Wei Lv, Shi-Xi Liu, Pei-Zhong Li, Jian-Bo Shi, Chun-Quan Ou

**Affiliations:** 1 Otorhinolaryngology Hospital, The First Affiliated Hospital, Sun Yat-sen University, Guangzhou 510080, China; fuqingl@mail.sysu.edu.cn (Q.-L.F.); entxgfess@163.com (G.X.); 2 State Key Laboratory of Organ Failure Research, Department of Biostatistics, Guangdong Provincial Key Laboratory of Tropical Disease Research, School of Public Health, Southern Medical University, Guangzhou 510515, China; duyue0524@163.com; 3 Department of Otorhinolaryngology, The First Affiliated Hospital, Xinjiang Medical University, Urumchi 830054, China; hzhang1106@163.com; 4 Department of Otorhinolaryngology, The First Affiliated Hospital, Nanjing Medical University, Nanjing 210029, China; chenglei@jsph.org.cn; 5 Department of Otorhinolaryngology, Union Hospital, Tongji Medical College, Huazhong University of Science and Technology, Wuhan 430022, China; ent-xh@163.com; 6 Department of Otolaryngology Head and Neck Surgery, China-Japan Union Hospital, Jilin University, Changchun 130033, China; zhudongdong2002@hotmail.com; 7 Department of Otolaryngology, Peking Union Medical College Hospital, Beijing 100032, China; lili20020615@sina.com; 8 Department of Otorhinolaryngology, West China Hospital of Medicine, Sichuan University, Chengdu 610041, China; liusx999@163.com; 9 Department of Otorhinolaryngology, Affiliated Huai’an First People’s Hospital, Nanjing Medical University, Huai‘an 223300, China; lpzent310@163.com

**Keywords:** asthma, China, prevalence, occupational and environmental risk factors

## Abstract

Objective: Asthma is one of the most common chronic diseases and associated with significant morbidity and mortality. However, few data on occupational and environmental risk factors of asthma are available, particularly in Asian adults. Based on a national cross-sectional survey, we assessed the prevalence and risk factors of asthma in Chinese adults. Methods: A total of 9974 participants aged 15 years and over in seven Chinese cities were selected using a stratified four-stage random sampling. All participants were interviewed face-to-face in their homes using a standardized self-administered questionnaire. Multivariate logistic regression analyses were adopted to determine various risk factors for asthma. Results: The prevalence of self-reported lifetime asthma was 2.46% among the entire adult population, 3.02% among males and 1.93% among females. The prevalence varied by age group, ethnicity, marital status, education, and floor space per person (*p* < 0.05). After adjusting for socio-demographic variables and smoking, we found independent occupational and environmental determinants of asthma, including a clearance-related job (OR = 2.28, 95%CI: 1.07–4.89), occupational exposure to industrial or occupational poisonous gas (OR = 4.21, 95%CI: 2.43–7.30), having large amounts of carpet in the workplace (OR = 2.61, 95%CI: 1.20–5.69) and using coal for cooking (OR = 2.65, 95%CI: 1.26–5.57). Conclusions: Asthma is a serious public health problem in China. Our study provides important updated information on the prevalence of asthma and its associated risk factors, which may help us better understand the epidemiology of asthma and prevent this disorder.

## 1. Introduction

Asthma is one of the most common chronic diseases and is associated with significant morbidity and mortality, particularly in children and the elderly [1]. Furthermore, asthma adds substantially to health care costs. For example, the economic cost of asthma in the U.S. was $59 billion in 2007, including direct health care costs of $53.1 billion and indirect costs or lost productivity of $5.9 billion [2]. These health and economic burdens are largely preventable with targeted interventions. An estimate of prevalence of asthma is required to assess the level of its associated disease burden, and identification of risk factors can help develop targeted intervention strategies for preventing asthma.

The prevalence of asthma has increased in Western countries in the last century, although it seems to have stabilized, and even decreased recently in some regions [3,4]. The risk of developing asthma varies widely between individuals and between communities, and the prevalence of asthma shows significant geographic and racial disparities [5,6]. In the U.S. in 2011, the average national prevalence of lifetime asthma and current asthma was estimated to be 13.3% and 8.5%, respectively [7]. The Global Allergy and Asthma European Network (GA^2^LEN) survey reported that the prevalence of asthma in European adults ranged from 5.2% to 16.8% in 19 centers in 12 countries [8]. The prevalence of asthma was 4.7% among the adult population in Turkey [9] and 12.4% in Brazil adolescents [10]. In Asia, a literature review by Song et al. [11] showed a prevalence of 0.7%–11.9% for adult asthma, generally lower than in Western countries. An increasing trend was identified in Japan, Korea, and Hong Kong [11]. Some single-city or single-province studies also indicate the prevalence of asthma is rapidly rising in mainland China [12,13]. The World Health Survey in 2002–2003 reported a prevalence of 0.2% and 1.4% for doctor-diagnosed asthma and clinical asthma, respectively, in Chinese populations [14]. In the past few decades, China has undergone dramatic social, economic, and environmental changes, which may be associated with potential changes in the epidemiology of asthma. Although a few studies have examined occupational and environmental risk factors of asthma in China [13,15], it is necessary to update the epidemiological characteristics of adult asthma based on a large-scale multi-city study in the general Chinese population. Based on a cross-sectional survey in seven Chinese metropolises, we assessed the prevalence of self-reported asthma and various socio-demographic, environmental, and occupational associated risk factors in Chinese adults.

## 2. Materials and Methods

### 2.1. Subjects

The study was carried out in 2012 in seven major Chinese cities, including Changchun, Beijing, Urumqi, Chengdu, Huaian, Wuhan, and Guangzhou, which are geographically representative cities in China with diverse climate and socio-economic levels. In each city, at least 500 households from eight communities were selected using a four-stage random sampling method. Considering that the prevalence of asthma and associated risk factors are significantly different among children compared with adults, this study only included adult participants (aged 15 years and over) from the selected households. The entire sampling process was carried out by the principle center using a unified protocol and a standardized computer-sampling program. The details of this sampling have been reported previously [16]. All participants were interviewed face-to-face in their own homes using a standardized questionnaire.

### 2.2. Ethics, Consent, and Permission

Informed consent was obtained from each subject before the interview. The study protocol was approved by the Ethics Committee of Sun Yat-sen University (the principle center, 201058), China.

### 2.3. Instruments and Outcome Measures

The questionnaire was modified from the Global Allergy and Asthma European Network (GA^2^LEN) questionnaire, which was validated and used in 12 countries [17]. The questionnaire consisted of socio-demographic characteristics, information on asthma and relevant diseases, life-style, and environmental factors. The interviewer explained the purpose of the investigation and the procedure. The respondents completed the questionnaire independently, though the trained interviewer was available to provide assistance for those who were illiterate or who needed clarification. Specifically, the respondents were asked “Have you ever been told by a physician or other health professional that you have asthma?” followed by “Have you had an asthma attack in the last year?” These two questions were used to identify lifetime asthma and current asthma attack, respectively [18]. Lifetime asthma was the primary outcome in this study. Participants were asked whether they engaged in a clearance-related or health care-related job and about their exposure to dust, poisonous gas, carpet or moldy or damp environments. Questions regarding participants’ methods of cooking and keeping warm in winter and the frequency of air conditioner use were also included in this questionnaire and considered to be important components of environmental exposure.

### 2.4. Statistical Methods

We estimated the prevalence of lifetime asthma and current asthma for the entire population, respectively. Due to the limited number of current asthma cases, further analyses of subgroups and risk factors were performed only for lifetime asthma. The chi-square test was used to compare the prevalence of lifetime asthma among subgroups stratified by gender, age, ethnicity, marital status, education, household income, and floor space. We fitted a full logistic regression model to simultaneously estimate the effects of smoking and second-hand tobacco smoke (SHS) and various environmental and occupational factors on asthma, after adjusting for age group, gender, ethnicity, marital status, education attainment, household income, and floor space per person. Then, a forward stepwise logistic regression model was fitted using a model selection criteria of entry (*p* < 0.05) and remove (*p* > 0.10) based on the likelihood ratio test. The effect estimates are presented as adjusted odds ratios (ORs) with 95% confidence intervals (95%CIs). All analyses were performed using SPSS 19.0 (IBM SPSS Software, Chicago, IL, USA) and STATA 12.0. (Stata Corp LP, College Station, TX, USA).

## 3. Results

Data were obtained from a total of 9974 adults for a response rate of 87%. 51.91% of the respondents were females. The mean age was 44.33 years (SD = 16.93 years) ranging from 15 to 98, and 13.18% was aged 65 years or older. A total of 245 respondents (2.46%) self-reported lifetime asthma, and 117 (1.17%) had an asthma attack in the last year. Among the 245 patients, 94 patients (38.37) were taking asthma medications, including nebulizers, inhalers, or pills. The prevalence of lifetime asthma and current asthma attack both ranged from 1.26% to 3.61% and 0.57% to 1.72% in seven cities, respectively (Table 1). Based on age and gender distribution of the Chinese population from the sixth national population census report, we calculated an overall age- and gender-standardized prevalence of 2.27% (98%CI: 1.98%–2.56%). The age-standardized prevalence was 2.77% (95%CI: 2.61%–2.93%) for males and 1.76% (95%CI: 1.40%–2.12%) for females.

Asthma was consistently more prevalent among males than females, with an overall OR of 1.58 (95%CI: 1.22–2.05) (Table 1 and Table 2). The prevalence of lifetime asthma increased with age (odds trend test for three age groups χ^2^ = 43.93, *p* < 0.001) with, approximately, a three-fold risk of asthma among those aged 60 and over compared with the 15–34 years group (OR = 3.04, 95%CI: 2.13–4.34). The minority population was at a higher risk of asthma than the Han (OR = 2.02, 95%CI: 1.32–3.09). Married individuals, particularly those who were widowed, had a higher prevalence of asthma than those who were not married. The prevalence of asthma also varied with socio-economic status. The prevalence tended to decrease with educational attainment (odds trend test for three age groups (χ^2^ = 8.10, *p* = 0.004). Floor space per person was another socio-economic determinant of asthma. The group above the median level (26.67 m^2^) had almost double the risk of asthma compared with the group below the median (OR = 1.90, 95%CI: 1.44–2.50). Those with a higher household monthly income per person seemed to have a slightly higher prevalence of asthma, though the difference between the two income groups was not significant (OR = 1.18, 95%CI: 0.90–1.56) (Table 2).

**Figure 1:**
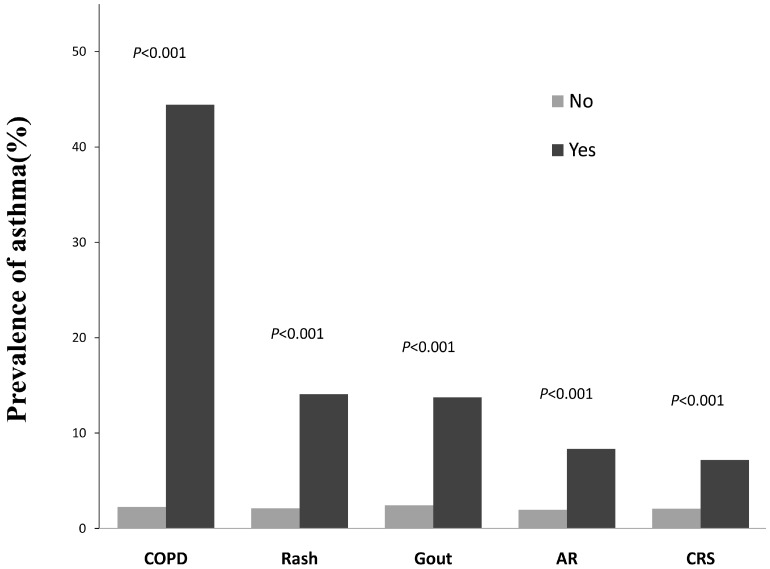
The prevalence of self-reported lifetime asthma in subjects with and without specific medical disorders, including chronic obstructive pulmonary disease (COPD), rash, gout, allergic rhinitis (AR), and chronic sinusitis (CRS).

We found that asthma was more common among those with a medical history of comorbidities than those without such comorbidities (*p* < 0.001). The asthma-associated morbidities included chronic sinusitis (CRS) (OR = 3.71, 95%CI: 2.74–5.02), allergic rhinitis (AR) (OR = 4.56, 95%CI: 3.42–6.07), gout (OR = 6.79, 95%CI: 4.25–10.85), rash (OR = 7.57, 95%CI: 5.27–10.86), and chronic obstructive pulmonary disease (COPD) (OR = 34.75, 95%CI: 19.99–60.42) (Figure 1).

The prevalence of current smoking was higher in asthma patients than in those without asthma (26.26% vs. 16.55%). After adjusting for age group, gender, ethnicity, marital status, education attainment, household income, and floor space per person, we observed a significant effect of current smoking on asthma (OR = 1.97, 95%CI: 1.36–2.85). An increased prevalence of asthma was also found among former-smokers with prevalence OR of 3.18 (95%CI: 1.80–5.64) compared with never-smokers. No significant effect of second-hand smoke was found (OR = 1.17, 95%CI: 0.83–1.65). Individuals who were overweight or underweight tended to have a higher risk of asthma than those with a normal body mass index (BMI), though the differences were not significant. We observed significant occupational and environmental risk factors associated with asthma. A clearance-related job increased the risk of asthma (OR = 2.28, 95%CI: 1.07–4.89). Those using coal for cooking had a much higher risk of asthma than those cooking with electric or gas appliances (OR = 2.65, 95%CI: 1.26–5.57). Occupational exposure to industrial or occupational poisonous gas (OR = 4.21, 95%CI: 2.43–7.30) and a large amount of carpet in the workplace were two other independent risk factors of asthma (OR = 2.61, 95%CI: 1.20–5.69) (Table 3).

## 4. Discussion

Previous epidemiological investigations of asthma have mainly focused on children, with a reported prevalence of 9% in Bangkok students aged 9.5 years [19], 17.7% among American children 5.5-years-old [20], and 1.1%–11.0% reported in previous studies for Chinese children aged 0–14 years [21]. There has been relatively little literature on adults. In this population-based multi-city study, we found a prevalence of self-reported lifetime asthma among Chinese adults of 2.46%. This prevalence is slightly higher than that previously reported among Chinese, such as 1.8% in Shanghai in 2007–2010 (1.8%) [12] and 1.4% in 2002–2003 from the World Health Survey [14], but much lower compared with a prevalence of 15% in the U.S. [7] and 5.2%–16.8% in 19 European centers [8], and is slightly higher than the prevalence of 1.9% reported in the Indian working population [22]. Notably, a prevalence of 12.8% in African populations has been reported in a systematic review, which is also much higher than our estimate in China. These comparisons suggest that the difference in the prevalence of asthma predominantly reflects differences between East and West, rather than between undeveloped and developed regions. Some literature has demonstrated that the prevalence of asthma and other allergic diseases (e.g., CRS, AR and rash) is lower in Asian countries than in Western countries [16,23]. Brim et al. reported a lower prevalence of current asthma in Asian Indian children (4.4%) than in American Indian/Alaska Native children (13.0%). Of greater interest, Shani et al. [24] found that the prevalence of asthma increases when immigrant populations move from undeveloped countries to new westernized environments. Furthermore, this prevalence gradually evolves to match the prevalence of asthma in the original Western population. This observation indicates that environmental factors play an important role in determining the geographic differences in asthma.

A GA^2^LEN review reported a consistently higher prevalence of asthma in boys than girls, though the risk of asthma was higher in females after puberty than males [25]. However, consistent with a previous survey in Shanghai, China [12], in this study of adults aged 15 and over, we observed that males were more likely to report asthma than females in six of the seven cities. The reason for this differential gender difference in China is unclear. A possible explanation is that compared with females, males had a higher smoking prevalence (35.4% vs. 2.8%), and were more likely to be occupationally exposed to dust (7.3% vs. 4.9%) and industrial or occupational poisonous gas (3.3% vs. 2.7%), reflecting gender differences in smoking prevalence to environmental and occupational region-specific risk factors. The evidence supporting socio-economic disparities in asthma prevalence are of great concern. We found that a lower educational level was associated with a higher asthma prevalence. Individuals reporting larger floor space per capita had a higher prevalence rate of asthma.

In addition to our study’s substantiation of the classical correlation between asthma and certain comorbidities, such as specific allergic diseases (CRS, AR, rash) and COPD, we also confirmed that active smoking is an important risk factor for asthma. The finding of a high prevalence of lifetime asthma among former smokers deserves further attention. Compared with current smoking, former smoking has been shown to have a stronger relationship to asthma in Saudi Arabian and European adults [8,26]. This may be due to inverse causality, namely that some individuals quit smoking because of the onset of respiratory symptoms or diagnosed asthma. In line with several studies that have shown obesity to also be a risk factor for asthma [27,28,29], we calculated a crude prevalence OR of 1.37 (95%CI: 1.02–1.84) for the high BMI group comparing with the normal BMI group. However, the adjusted OR was no longer significant after adjusting for socio-demographic factors and smoking, suggesting that obesity may be not an independent risk factor for asthma in Chinese adults.

Exposure to wood smoke has been identified as a risk factor of asthma in Chinese rural communities and some underdeveloped regions where using wood for cooking is very prevalent [15,30,31]. However, a multivariate analysis of Indian adults revealed that cooking fuel type (clean or unclean) was not an independent factor associated with asthma [29]. In this study in seven large cities, we found using coal for cooking was an independent indoor environmental determinant of asthma, but a significant effect was not observed for wood smoke, probably because of the low prevalence of using wood (2.21%). Using coal for cooking adversely affects indoor air quality, and air pollution has been linked to an increased prevalence of asthma as well as asthma-related morbidity and mortality in both children and adults [32,33]. Having large amounts of carpet in the work place was also found to be associated with increased asthma prevalence, probably due to increased exposure to allergens and dust [34]. Additionally, occupational exposure to industrial or occupational poisonous gas remarkably increased the risk of asthma. Therefore, supporting the use of protective masks in the work place should be encouraged.

There are some limitations associated with this study. First, the reliability and validity of self-reported asthma was not systematically tested. A review reported a mean sensitivity for self-reported asthma of 68% and a mean specificity of 94% when validated in relation to a clinical diagnosis of asthma [35]. Thus, asthma prevalence may potentially be underestimated when based on self-reported data. All exposure measures were also self-reported, which may lead to recall bias and misclassification. We used a standardized questionnaire modified according to GA^2^LEN to reduce this potential bias. Additionally, occupational exposure was classified into “yes” and “no” in our analyses, although we collected information about the species of dust and industrial or occupational poisonous gas and the intensity and duration of exposure. However, we did not find that the effects statistically increased with the exposure level, probably because of a small sample size of subgroups by exposure levels in the general population. Further data of intensity and duration of the exposure collected from specific occupational workers would identify a potential dose-response relationship. Furthermore, the effects were estimated after adjusting for demographic and socioeconomic characteristics and smoking, while there may be some other potential confounders, such as alcohol drinking and ambient air pollution exposure, which were not considered in this study. We found the prevalence of asthma was much higher in those with COPD, rash, gout, allergic rhinitis and CRS. We cannot avoid Berkson’s bias since the co-existence with another chronic condition (i.e., COPD) may increase the risks of being diagnosed with asthma. Lastly, the randomness of sampling and the representativeness of the subjects were verified by the comparability of demographic structure (e.g., age, sex, and education level) between the sample and the sixth national census data in each city [16]. However, the participants were from seven major Chinese cities where the prevalence may be different from that in small cities and rural regions, the reported prevalence of 2.46% in this study may be a biased national estimate in China.

## 5. Conclusions

This population-based cross-sectional investigation revealed that asthma is an important public health problem in China. An estimated 26.68 million adults are affected by asthma in mainland China, although the prevalence of 2.46% is relatively lower than in Western countries. We observed substantial geographic variations in the prevalence of asthma and identified disproportionally affected populations, including males, the elderly, smokers, and those with a low educational level. Our study provides insight into the excess risk of asthma due to environmental and occupational factors, including using coal for cooking, occupational exposure to industrial or occupational poisonous gas, and having large amounts of carpet in the workplace. Our findings are useful for assessing the social and economic burden of asthma.

## Figures and Tables

**Table 1:** Prevalence of asthma in different cities.

City	Prevalence of Lifetime Asthma (%)			Prevalence of Current Asthma Attack (%)		
	Male	Female	Total	Male	Female	Total
Changchun	1.30 (9/692)	1.57 (11/699)	1.44 (20/1391)	0.08 (4/692)	1.14 (8/699)	0.86 (12/1391)
Urumqi	3.91 (28/717)	2.99 (22/735)	3.44 (50/1452)	2.09 (15/717)	1.36 (10/735)	1.72 (25/1452)
Beijing	1.65 (11/668)	0.92 (7/763)	1.26 (18/1431)	1.05 (7/668)	0.26 (2/763)	0.63 (9/1431)
Huai’an	4.80 (31/646)	1.46 (11/752)	3.00 (42/1398)	2.17 (14/646)	0.93 (7/741)	1.50 (21/1398)
Wuhan	3.44 (25/727)	2.90 (21/725)	3.17 (46/1452)	1.79 (13/727)	1.38 (10/725)	1.58 (23/1452)
Chengdu	4.57 (30/657)	2.81 (22/782)	3.61 (52/1439)	1.83 (12/657)	0.90 (7/782)	1.32 (19/1439)
Guangzhou	1.60 (11/688)	0.83 (6/723)	1.20 (17/1411)	0.87 (6/688)	0.28 (2/723)	0.57 (8/1411)
Total	3.02 (145/4795)	1.93 (100/5179)	2.46 (245/9974)	1.48 (71/4795)	0.89 (46/5179)	1.17 (117/9974)

**Table 2:** Comparisons of the prevalence of lifetime asthma among different socio-demographic groups.

Factors	Groups	Responders n (Proportion, %)	Asthma n (Prevalence, %)	*p*
Gender	Female	5169 (51.91)	100 (1.93)	<0.001
	Male	4787 (48.08)	145 (3.02)	
Age group	15–34 years	3136 (29.53)	43 (1.37)	<0.001
	35–59 years	4030 (37.95)	88 (2.18)	
	≥60 years	2809 (26.45)	114 (4.06)	
Ethnicity	Han	8654 (94.60)	220 (2.54)	0.001
	Minority	494 (5.40)	25 (5.06)	
Marital status	Married	7662 (77.02)	195 (2.55)	<0.001
	Divorced	152 (1.53)	4 (2.63)	
	Widowed	427 (4.29)	22 (5.15)	
	Unmarried	1707 (17.16)	22 (1.29)	
Education attainment	Illiterate/primary	1123 (11.26)	38 (3.38)	0.006
	Secondary school	2042 (20.48)	55 (2.69)	
	High school	3380 (33.91)	82 (2.43)	
	College	3205 (32.15)	68 (2.12)	
	Master or above	219 (2.20)	1 (0.46)	
Household monthly income per person	<RMB 3000	7264 (73.10)	171 (2.35)	0.240
	RMB 3000+	2673 (29.90)	74 (2.77)	
Floor space per person (m^2^)	Below median (26.67)	4396 (51.41)	81 (1.84)	<0.001
	Above median	4155 (48.59)	143 (3.44)	

**Table 3:** The effects of environmental and occupational factors on lifetime asthma.

	Asthma n (Proportion, %)	Non-Asthma n (Proportion, %)	Adjusted Odds Ratio (95%CI) *	
			Full Model	Stepwise Model
**Clearance-related job**	13 (5.46)	253 (2.67)	1.77 (0.77,4.08)	2.28 (1.07,4.89)
**Health care-related job**	16 (6.72)	516 (5.45)	1.62 (0.83,3.14)	
**Occupational exposure to dust**	34 (13.93)	604 (6.25)	1.37 (0.77,2.46)	
**Occupational exposure to industrial or occupational poisonous gas**	29 (11.84)	281 (2.91)	3.49 (1.91,6.38)	4.21 (2.43,7.30)
**Large amounts of carpet at home**	24 (10.67)	561 (6.73)	0.67 (0.34,1.33)	-
**Large amounts of carpet at work place**	13 (5.78)	126 (1.51)	2.99 (1.24,7.21)	2.61 (1.20,5.69)
**Cooking method at home**				
Electric or gas	229 (93.47)	9299 (96.23)	1	1
Coal	12 (4.90)	146 (1.51)	2.11 (0.85,5.25)	2.65 (1.26,5.57)
Firewood	4 (1.63)	218 (2.26)	1.48 (0.40,5.39)	1.36 (0.42,4.42)
**Maintaining warmth in winter**				
Electric or centralized heating	175 (94.59)	6302 (96.42)	1	1
Coal	8 (4.32)	134 (2.05)	1.24 (0.47,3.27)	
Firewood/charcoal	2 (1.08)	100 (1.53)	0.37 (0.08,1.74)	
**Exposure to mouldy or damp environments**				
Never	175 (78.78)	7037 (84.34)	1	1
Occasionally	40 (17.78)	1063 (12.74)	1.48 (0.93,2.35)	
Frequently or everyday	10 (4.44)	244 (2.92)	1.56 (0.67,3.63)	
**Smoking status**				
Non-smokers	156 (69.93)	7813 (80.55)	1	1
Former smokers	24 (9.83)	282 (2.91)	2.64 (1.42,4.90)	3.18 (1.80,5.64)
Current smokers	64 (26.23)	1605 (16.55)	1.63 (1.06,2.50)	1.97 (1.36,2.85)
Second-hand smoke	110 (45.08)	3696 (38.24)	1.17 (0.83,1.65)	
**Body mass index(BMI) (reference group)**				
Normal: 18.5–25 kg/m^2^	141 (62.67)	5727 (68.81)	1	1
Low: <18.5 kg/m^2^	17 (7.56)	611 (7.34)	1.41 (0.73,2.72)	
High: >25 kg/m^2^	67 (29.78)	1985 (23.85)	1.16 (0.81,1.66)	

* All effects were estimated after adjusting for age group, gender, ethnicity, marital status, education attainment, household income, and floor space per person, and other co-variables in this table.

## Abbreviations

OR: odds ratio
CI: confidence interval
GA^2^LEN: The Global Allergy and Asthma European Network
CRS: chronic sinusitis
AR: allergic rhinitis
COPD: chronic obstructive pulmonary disease
BMI: body mass index
